# IgG4 related pleural disease: Recurrent pleural effusion after COVID‐19 vaccination

**DOI:** 10.1002/rcr2.1026

**Published:** 2022-09-18

**Authors:** Saria Tasnim, Ola Al‐Jobory, Ahmad Hallak, Taru Bharadwaj, Manish Patel

**Affiliations:** ^1^ Department of Internal Medicine Texas Tech University Health Sciences Center Amarillo Texas USA; ^2^ Department of Vascular Medicine Ochsner Medical Center New Orleans Louisiana USA

**Keywords:** COVID‐19 vaccine, IgG4‐related disease, IgG4‐related lung disease, Pfizer vaccine, pleural effusion

## Abstract

IgG4‐related disease is characterized by a systemic fibroinflammatory process associated with substantial infiltration by plasma cells with IgG4 in the organs. Our patient presented with pleural effusion, and was diagnosed with IgG4‐related lung disease (IgG4‐RLD) after he received two doses of the Pfizer COVID‐19 vaccine. The patient developed dyspnea and hypoxia 2 weeks after receiving the second dose of the Pfizer COVID‐19 vaccine. CT scan revealed left pleural effusion which was drained. However, the effusion recurred requiring thoracoscopic drainage, placement of an indwelling catheter, and decortication with biopsy. IgG4 serum level was 268 mg/dl and pathology revealed pleural fibrosis, lymphoplasmacytic infiltrates, and increased IgG4‐positive plasma cells with no malignant cells leading to a diagnosis of IgG4‐RLD. Although COVID vaccine‐related IgG4‐RLD is a novel finding, having a high degree of suspicion following vaccination is always important for early diagnosis and effective treatment.

## Graphical abstract

We report the case of a patient who presented with pleural effusion, and was diagnosed with IgG4‐related lung disease (IgG4‐RLD) after he received two doses of the Pfizer COVID‐19 vaccine. Although COVID vaccine‐related IgG4‐RLD is a novel finding, having a high degree of suspicion following vaccination is always important for early diagnosis and effective treatment.

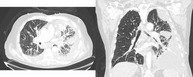

## INTRODUCTION

IgG4‐related disease (IgG‐RD) is a poorly understood inflammatory disease. It is a systemic fibroinflammatory process associated with substantial infiltration by plasma cell with IgG4. Since the COVID era, great effort went into finding a vaccine to decrease the impact of the virus, several companies achieved their goal by finding a vaccine, including the Pfizer vaccine which is a segment of mRNA of the virus wrapped with lipid nanoparticles to activate the immune system.^1^ Although, side effects related to the Pfizer vaccine are benign, very rarely, it can lead to IgG‐RD.^2^

## CASE REPORT

**FIGURE 1:**
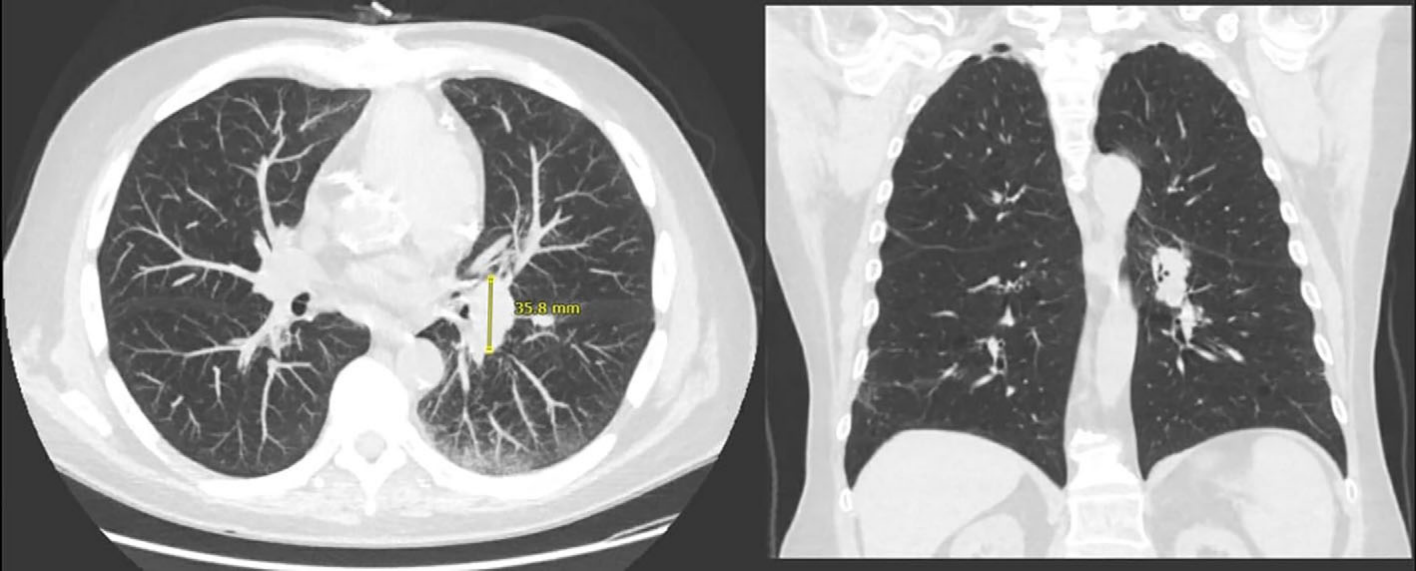
Transverse and coronal view of chest CT showing pulmonary nodule prior to COVID vaccine

Our patient is a 71‐year‐old male with hypertension, coronary artery disease, prostate cancer and chronic obstructive pulmonary disease with a 50‐pack year smoking history. The patient started being followed in the pulmonology department after a screening chest CT revealed left hilar fullness and peri‐fissural nodules, the largest of which measured 0.4 cm (Figure 1). Repeat CT 2 years later showed unchanged nodules, with two new nodules measuring 0.4 cm. At this point, he elected not to continue surveillance.

Two years later, he received a first dose of the Pfizer COVID‐19 vaccine and visited urgent care after a week with wheezing and dyspnea which improved with albuterol. However, he returned to clinic with dyspnea 2 weeks after receiving the second dose of the vaccine. He underwent pulmonary function testing which revealed a mixed obstructive and restrictive pattern with reduced DLCO. Six‐minute walk test revealed hypoxia requiring 3 L oxygen.

**FIGURE 2:**
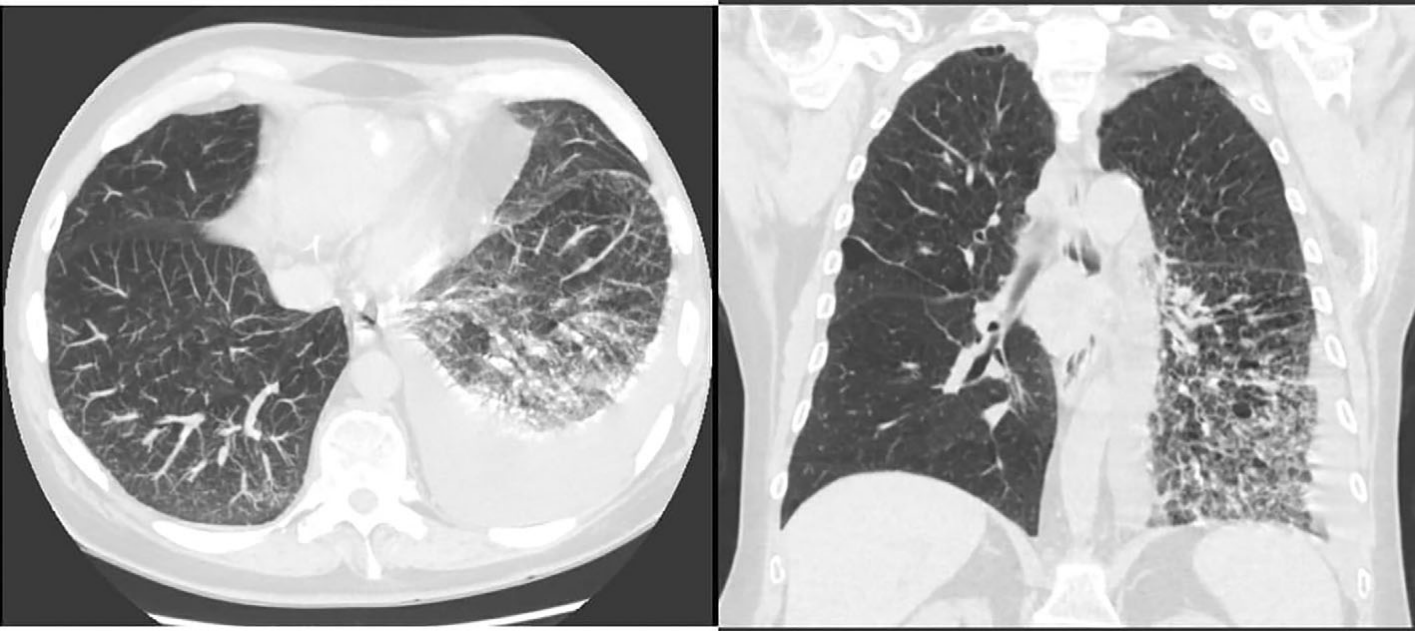
Transverse and coronal view of chest CT showing left‐sided pleural effusion with atelectasis

Chest CT revealed new left pleural effusion with atelectasis (Figure 2). 1.4 L straw‐coloured exudative fluid was removed by thoracentesis which was negative for malignant cells. Summary of the laboratory, pulmonary function test and pleural fluid analysis of our case are shown in Table 1.

**TABLE 1:** Summary of the laboratory, pulmonary function test and pleural fluid analysis of our case

Serum								
Total protein, g/dl	Albumin, g/dl	Lactate dehydrogenase (LDH), U/L	Creatinine, mg/dl	Glucose, mg/dl	Total bilirubin, mg/dl	Aspartate amino‐transferase (AST), U/L	Alanine amino‐transferase (ALT), U/L	IgG4, mg/dl
7.1	2.3	190	0.9	83	0.5	31	12	268

Pleural fluid										
Colour and appearance	White blood cell (WBC)/ml	Segmented cell	Lymphocyte	Monocyte/macrophage	Eosinophil	Lactate dehydrogenase (LDH), U/L	Triglyceride, mmol/L	Albumin, g/dl	Total protein, g/dl	Cholesterol, mg/dl
Yellow and Hazy	3507	4%	90%	5%	1%	168	16	1.7	5.8	37

Pulmonary function test									
Forced vital capacity (FVC)	Forced expiratory volume in 1 s (FEV1)	FEF 25%–75%	Peak expiratory flow (PEF)	FEV1/FVC	Total lung capacity (TLC)	Residual volume (RV)	RV/TLC	Diffusion capacity of the lungs for carbon monoxide (DLCO)	DLCO/Alveolar volume (DL/VA)
80.9%	68.9%	47.4%	64.7%	65	77.7	74.1	36.45	40.4%	50.4%

His symptoms initially improved but worsened again. Repeat CT scan 10 days later revealed large left pleural effusion. He was referred to thoracic surgery and underwent left thoracoscopy with drainage of 2.5 L of pleural fluid followed by pleural biopsy and chemical pleurodesis with insertion of an indwelling tunnelled pleural catheter. Pleural biopsy revealed chronic organizing pleuritis with lymphoid hyperplasia and reactive mesothelial hyperplasia.

**FIGURE 3:**
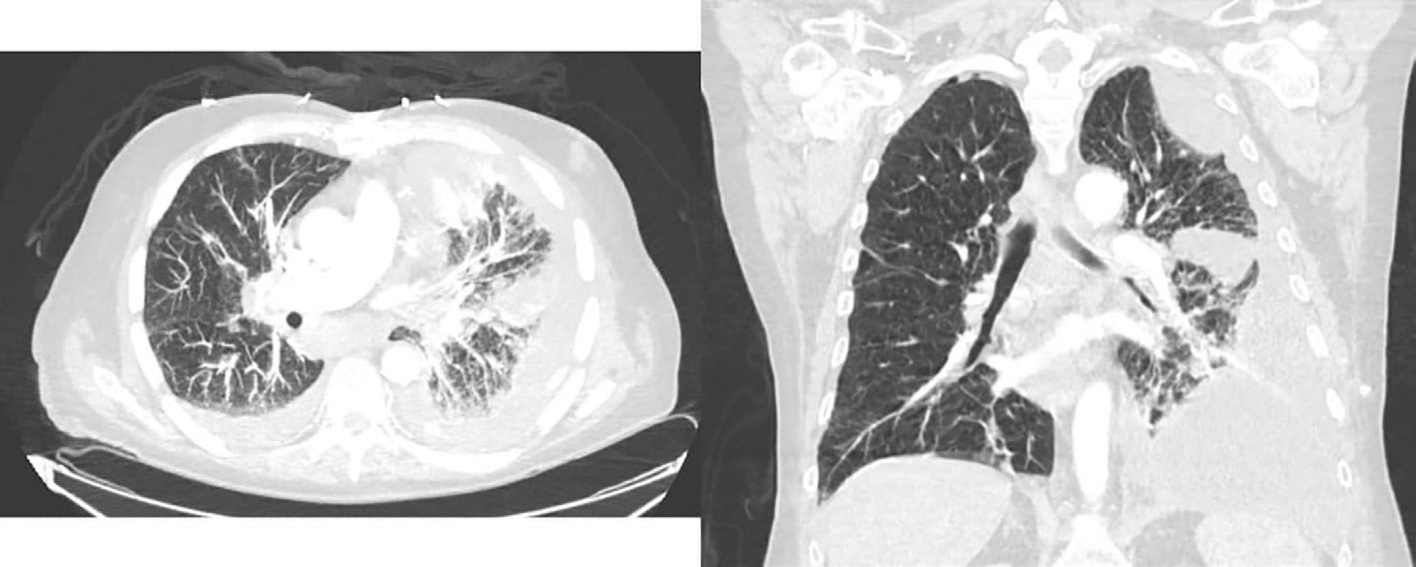
Transverse and coronal view of chest CT showing loculated left‐sided pleural effusion

The pleural catheter continued to drain for 3 months. Eventually, it stopped draining but oxygen requirement increased to 5 L. Repeat CT scan revealed loculated pleural effusions (Figure 3). Thoracentesis was attempted but only 40 ml bloody fluid was drained.

Thoracoscopy revealed multiloculated pleural effusions with significant visceral pleural thickening and the patient underwent partial decortication. Pathology report revealed pleural thickening and fibrosis with surrounding pulmonary parenchyma revealing diffuse alveolar damage, lymphoplasmacytic infiltrates, and increased IgG4‐positive plasma cells with fibrinous adhesions. At this time, blood IgG4 level was 268 mg/dl. Autoimmune, malignant and genetic conditions with similar presentation were excluded. The patient was diagnosed with IgG4‐RD affecting the lungs and pleura, non‐pulmonary involvement was not found. He was referred to the pulmonology clinic for further management but he was lost to follow‐up.

## DISCUSSION

Pfizer‐BioNTech COVID‐19 vaccine is given intramuscularly to individuals aged 5 years or older in two doses 3 weeks apart. Local and systemic adverse effects are usually mild. There was only one case report of recurrence of IgG4‐related nephritis following second dose of Pfizer COVID‐19 vaccine.^2^ This is the first reported case of IgG4‐related pulmonary and pleural disease after Pfizer COVID‐19 vaccine.

The pathogenesis of IgG4‐RLD remains unclear. Autoimmunity is a trigger with a key role of T helper‐2 cell.^3^, ^4^ The vaccine promotes potent immune response by stimulating robust antigen‐specific T‐cell responses including T follicular helper cells, and potent germinal centre B‐cell responses leading to neutralizing antibody production. The antibodies trigger autoimmune reaction secondary to molecular mimicry. The SARS‐CoV‐2 spike protein shares homology with several human proteins including human alveolar surfactant‐related proteins, which may be subject to off‐target immune attack after vaccination^3^ and subsequent pulmonary pathology.

Four patterns are observed in IgG4‐RLD, which include mediastinal, parenchymal, pleural, and airway involvement. Mediastinal and hilar lymphadenopathy are the commonest patterns. Parenchymal involvement was further sub‐categorized into: (I) solid nodular type; (II) round ground‐glass opacity type; (III) alveolar interstitial type; and (IV) broncho‐vascular type. Pleural effusion or nodules is uncommon. Airway involvement is rare, including tracheobronchial stenosis and traction bronchiectasis.^4^ In our patient, there was pleural effusion with atelectasis.

The definite diagnosis of IgG4‐RLD relies on histopathological analysis. The key morphological features are dense lymphoplasmacytic infiltrates with collagenised fibrosis and active fibroblastic proliferation.^4^ Patients who fulfil the following three criteria are diagnosed as having IgG4‐RD: consistent organ involvement or damage; serum IgG4 level of >135 mg/dl; and histopathology showing marked lymphoplasmacytic infiltration, fibrosis. Our case fulfilled all three comprehensive diagnostic criteria and involved lung; therefore, the diagnosis of IgG4‐RLD was made.

The mainstay treatment for IgG4‐RLD is corticosteroid therapy, and most patients have favourable response in 2 weeks. The usual dose for oral prednisone is between 0.6 and 1 mg/kg/day, and then gradually tapered every 2–4 weeks to maintain a dose of 2.5–5 mg/day and then discontinued within 3 years. For steroid‐refractory patients, immunosuppressants or anti CD20 antibody can be considered.^5^

As the worldwide COVID‐19 vaccination continues to accelerate, it is probable to see more autoimmune events. However, due to reporting bias and pre‐existing undiagnosed autoimmune lung disease, true incidence of IgG4‐RLD will be difficult to determine. As multiple doses of vaccines are now being offered, close observation will be needed. Anyone with rapidly progressive lung or pleural disease after COVID 19 vaccine, more aggressive approach for diagnosis is recommended so diseases such as IgG4‐RLD cannot be missed and prompt treatment can be started to mitigate this disease.

## AUTHOR CONTRIBUTION

The authors listed above were involved in taking consent from the patient, writing the manuscript and reviewing the manuscript.

## CONFLICT OF INTEREST

None declared.

## ETHICS STATEMENT

The authors declare that appropriate written informed consent was obtained for the publication of this manuscript and accompanying images.

## Data Availability

The data that support the findings of this study are available from the corresponding author upon reasonable request.
